# Clinical Study of a Wearable Remote Rehabilitation Training System for Patients With Stroke: Randomized Controlled Pilot Trial

**DOI:** 10.2196/40416

**Published:** 2023-02-23

**Authors:** Liquan Guo, Jiping Wang, Qunqiang Wu, Xinming Li, Bochao Zhang, Linfu Zhou, Daxi Xiong

**Affiliations:** 1 School of Biomedical Engineering (Suzhou) Division of Life Sciences and Medicine University of Science and Technology of China Suzhou China; 2 Suzhou Institute of Biomedical Engineering and Technology Chinese Academy of Sciences Suzhou China; 3 Department of Rehabilitation Medicine Tangdu Hospital Airforce Medicine University Xi‘an China; 4 Department of Rehabilitation Medicine Xi’an Gaoxin Hospital Xi'an China; 5 Department of Respiratory and Critical Care Medicine The First Affiliated Hospital Nanjing Medical University Nanjing China

**Keywords:** remote rehabilitation, wearable devices, human-computer interaction, rehabilitation training, stroke

## Abstract

**Background:**

In contrast to the large and increasing number of patients with stroke, clinical rehabilitation resources cannot meet their rehabilitation needs. Especially for those discharged, ways to carry out effective rehabilitation training without the supervision of physicians and receive guidance from physicians remain urgent problems to be solved in clinical rehabilitation and have become a research hot spot at home and abroad. At present, there are many studies on home rehabilitation training based on wearable devices, Kinect, among others, but these have disadvantages (eg, complex systems, high price, and unsatisfactory rehabilitation effects).

**Objective:**

This study aims to design a remote intelligent rehabilitation training system based on wearable devices and human-computer interaction training tasks, and to evaluate the effectiveness and safety of the remote rehabilitation training system for nonphysician-supervised motor rehabilitation training of patients with stroke through a clinical trial study.

**Methods:**

A total of 120 inpatients with stroke having limb motor dysfunction were enrolled via a randomized, parallel-controlled method in the rehabilitation institutions, and a 3-week clinical trial was conducted in the rehabilitation hall with 60 patients in the experimental group and 60 in the control group. The patients in the experimental group used the remote rehabilitation training system for rehabilitation training and routine clinical physical therapy (PT) training and received routine drug treatment every day. The patients in the control group received routine clinical occupational therapy (OT) training and routine clinical PT training and routine drug treatment every day. At the beginning of the training (baseline) and after 3 weeks, the Fugl-Meyer Motor Function Rating scale was scored by rehabilitation physicians, and the results were compared and analyzed.

**Results:**

Statistics were performed using SAS software (version 9.4). The total mean Fugl-Meyer score improved by 11.98 (SD 8.46; 95% CI 9.69-14.27) in the control group and 17.56 (SD 11.65; 95% CI 14.37-20.74) in the experimental group, and the difference between the 2 groups was statistically significant (*P*=.005). Among them, the mean Fugl-Meyer upper extremity score improved by 7.45 (SD 7.24; 95% CI 5.50-9.41) in the control group and 11.28 (SD 8.59; 95% CI 8.93-13.62) in the experimental group, and the difference between the 2 groups was statistically significant (*P*=.01). The mean Fugl-Meyer lower extremity score improved by 4.53 (SD 4.42; 95% CI 3.33-5.72) in the control group and 6.28 (SD 5.28; 95% CI 4.84-7.72) in the experimental group, and there was no significant difference between the 2 groups (*P*=.06). The test results showed that the experimental group was better than the control group, and that the patients’ motor ability was improved.

**Conclusions:**

The remote rehabilitation training system designed based on wearable devices and human-computer interaction training tasks can replace routine clinical OT training. In the future, through medical device registration certification, the system will be used without the participation of physicians or therapists, such as in rehabilitation training halls, and in remote environments, such as communities and homes.

**Trial Registration:**

Chinese Clinical Trial Registry ChiCTR2200061310; https://tinyurl.com/34ka2725

## Introduction

Stroke is a disease of cerebral blood circulation disorder and brain tissue function and structural damage caused by cerebral vascular obstruction or rupture. It is the third leading cause of death and the second leading cause of disability worldwide. The high disability rate increases economic burden and mental pressure on both society and families [1]. According to the “Report on Stroke Prevention and Treatment in China 2021” [2], in 2020, the standardized prevalence of stroke among people aged over 40 years in China was 2.61%, the incidence rate was 505.23/100,000, and there were about 17.8 million patients with stroke. In addition, 3.4 million new patients are diagnosed with stroke each year in China, compared with approximately 13.7 million annually worldwide.

According to statistics, about 70%-85% of patients with first-time stroke have limb motor dysfunction, which seriously affects the quality of life and brings a heavy burden for the family and society. Timely and effective rehabilitation training can help them restore certain motor functions [3]. However, compared with the large and increasing number of patients with stroke, the resources of rehabilitation medical care are very limited. Therefore, rehabilitation training with the participation of nonrehabilitation physicians or therapists, especially remote and home-based rehabilitation, has received increasing attention.

According to some studies [4,5], the effect of rehabilitation training in a remote environment is comparable to or even better than that in a hospital environment. Relevant studies show [6-8] that sustained and effective remote rehabilitation can activate the neuroplasticity of patients with stroke and greatly improve the rehabilitation effect. Remote rehabilitation training can save medical resources, promote the motor function of patients, and improve the rehabilitation level after discharge in view of poor compliance of discharged patients [9-11]. Indeed, patient adherence and acceptability of rehabilitative practices need to be actively enhanced, overcoming pitfalls due to motor (eg, endurance), nonmotor (eg, fatigue, pain, dysautonomic symptoms, and motivational), and cognitive deficits [12].

Active and effective rehabilitation with nonphysician involvement, such as remote and home rehabilitation, and uploading training data and results to physicians for analysis and guidance are effective solutions to the problems of lack of clinical rehabilitation resources and poor adherence of discharged patients and are hot spots of international research; however, they still face many challenges.

Currently, to carry out effective rehabilitation training with nonphysician involvement, 2 main technical support solutions are proposed for training data acquisition and human-computer interaction control around application scenarios such as patient limb movements [13-15], activity detection [16-18], and motion recognition [19-22].

The first is vision-based solution, such as using a depth camera or Kinect. Placidi et al [23] designed a simple motion analysis system based on the use of a depth camera and a 3D real-time model of the human body. Their experimental results showed no significant differences in more than 95% of the data. However, the experiment could not achieve the rehabilitation training goals for fine motor movements and was not suitable for patients with severe disabilities. Webster and Celik [24] summarized the application of Kinect in geriatric care and stroke rehabilitation, based on which it was pointed out that the current application should be simulated toward the real situation, there was the need to capture obscuring movements, and in addition, the Kinect application is vulnerable to the spatial environment.

The second is a wearable sensor-based solution that integrates inertial sensors such as accelerometers to assess functional activities related to patient mobility in terms of type, intensity, time, and quality of the activity. Rau et al [25] developed a triaxial accelerometer-based remote assessment system for acquiring kinematic data on upper extremity anterior extension movements in patients with stroke. Spearman analysis showed a strong correlation between this remote assessment system and standard kinematics. However, the experiment was only for upper extremity movements and required an expert on hand for guidance. Yang et al [14] proposed a stroke rehabilitation system combining inertial measurement sensors with physiological sensors, with an average recognition accuracy of 96.20% for hand gesture movements. However, this study only focused on identifying patient-specific movements and the training and validation data were from the same patients.

In addition, most studies have been conducted in clinical settings under physician supervision, and there is a lack of studies on rehabilitation training without physician supervision and validation in standardized clinical trials [26,27]. Therefore, to conduct effective rehabilitation training without physician supervision for use in remote and home settings, wearable devices based on the inertial measurement unit (IMU) and flex sensor are designed to be worn on the affected limb. Patients undergo interactive rehabilitation training based on standard training videos combined with human-computer interaction games. The feasibility, efficacy, and safety of the system are evaluated by conducting a 120-case parallel-controlled, 2-center clinical trial.

## Methods

### Overview of the System Framework

**Figure 1:**
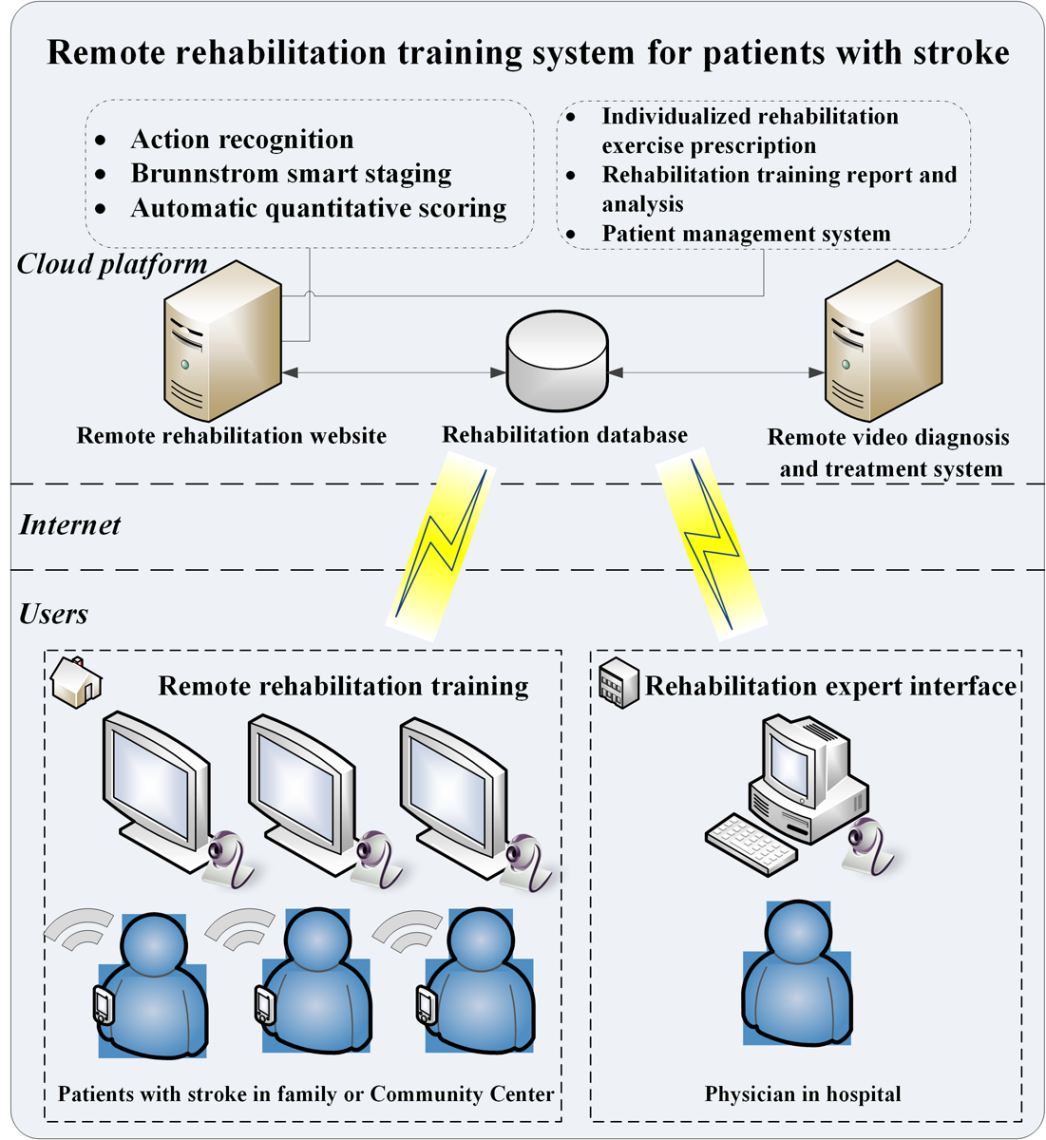
Remote rehabilitation training system for patients with stroke.

In this study, we designed wearable devices such as rehabilitation training gloves and upper and lower limb rehabilitation training modules. Further, a remote rehabilitation system integrating training equipment hardware, man-machine communication training games, rehabilitation training software, a remote rehabilitation management platform, and a mobile app were developed. The overall architecture of the system is shown in Figure 1. The system consists of 3 parts, the patients with stroke side, the physician side, and the cloud server. Through the remote server, the rehabilitation physician in the hospital can view, analyze, and guide the patients’ rehabilitation training remotely in the training hall, community, and home, and prescribe new rehabilitation exercises for the patients. Through this system, patients can perform rehabilitation training without physician involvement.

### Training Equipment Hardware

The wearable remote rehabilitation training equipment mainly includes the IMU modules for upper and lower limb rehabilitation training, rehabilitation training gloves for hand rehabilitation training, and Zigbee wireless receiver, among others. There are 2 IMU modules containing 9-axis motion sensors, including a 3-axis accelerometer, a 3-axis angular velocity meter, and a 3-axis magnetometer. The 2 IMU sensors are fixed to the upper and lower arms, respectively, by straps during upper limb training, and to the thigh and calf, respectively, during lower limb training. The rehabilitation glove contains 1 IMU and 5 flex sensors inside to monitor the movement of the wrist and individual fingers. The gloves are designed for left and right hands, with large, medium, and small sizes available to suit different patients.

The sensor is fixed on the affected side by the patients or their family according to the instructions and the wearing process is not complicated. In addition, the sensor works in headless mode, and its current position is automatically defined as the initial position through coordinate transformation at the beginning of each movement, so the deviation of the placement position does not affect the rehabilitation training.

Each wearable device has a 400-mAh battery and a power consumption of 20 mAh, with a full charge meeting the rehabilitation training for about 20 hours. Patients can train 2 times a day for half an hour each time, so the wearable devices can be used continuously for 10 days with a full charge. Each sensor was previously networked through the ZigBee2007 wireless communication protocol, which is very convenient for expansion and synchronous data collection. The sampling rate of each wearable device is 30 times per second, which is sufficient for rehabilitation training exercise data collection and analysis.

**Figure 2:**
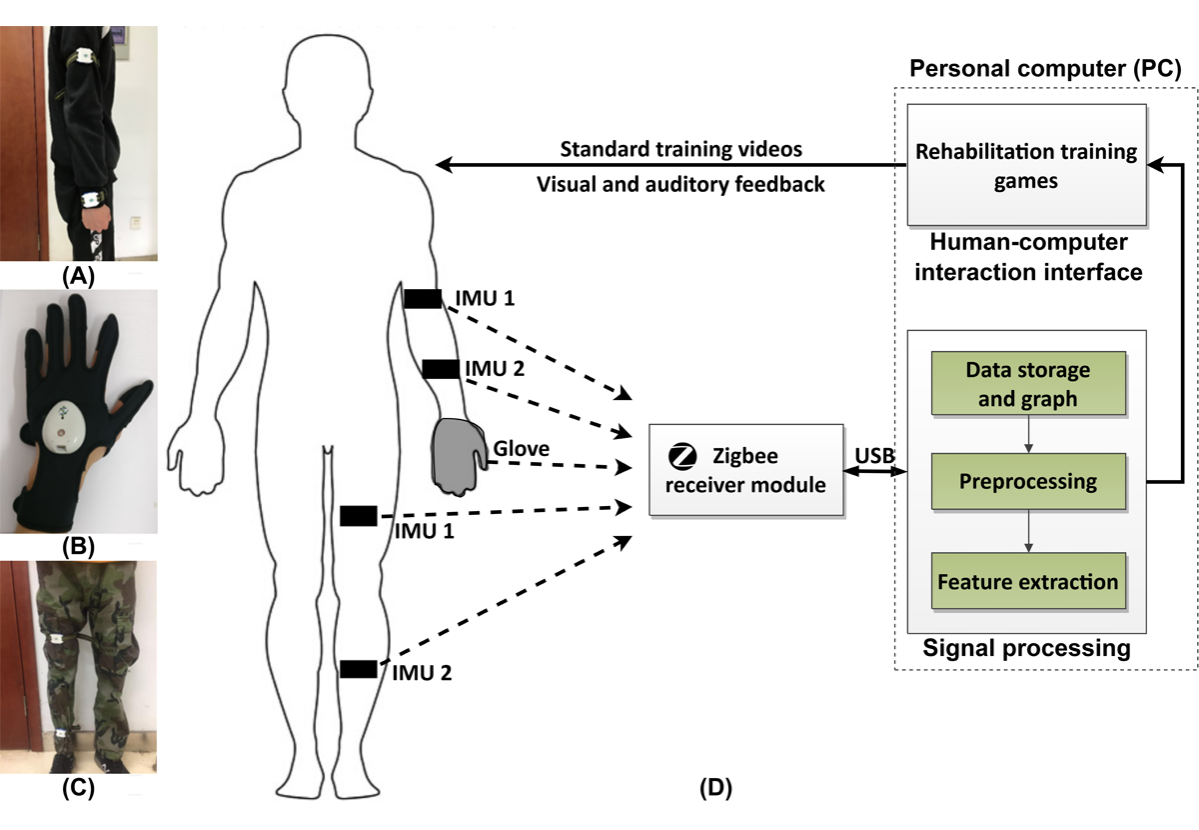
Rehabilitation training process based on wearable devices and interactive games. (A) Instructions for wearing upper limbs; (B) Instructions for wearing gloves; (C) Instructions for wearing lower limbs; (D) Human-computer interaction rehabilitation training process. IMU: inertial measurement unit.

The rehabilitation training process based on the wearable device and interactive game is shown in Figure 2. Panels A-C are the schematic diagrams of the wearable device worn on the affected upper limb, hand, and lower limb, respectively, while D is the schematic diagram of the human-computer interaction rehabilitation training process. Patients wear the wearable device according to the instruction manual and undergo rehabilitation training according to the standard training video on the software. The sensor collects the rehabilitation training data, receives them through the Zigbee wireless receiver, and transmits them to the computer through USB. The rehabilitation training software on the PC side collects, stores, and displays data; improves signal quality through preprocessing such as sliding filtering; and then extracts patient motion features. The system controls rehabilitation training games through motion features, conducts human-computer interaction training for patients, and provides video and auditory feedback to patients to improve their enthusiasm for rehabilitation training.

Under the advice of the rehabilitation physician and according to the characteristics of rehabilitation training movements, the motion features extracted by the system mainly include motion amplitude, direction, dynamic energy, motion smoothness, and motion force size, as shown in Table 1. The training difficulty is set according to the Fugl-Meyer score of the patient at the time of enrollment, and the system automatically adjusts the difficulty of the next training according to the previous training, with different features selected for different rehabilitation training movements and difficulty levels. For example, the simple mode of the Bobath handshake training uses AMP (amplitude) as the training game control parameter, while the hard mode collects all 5 features (amplitude, mean value, root-mean-square, JERK, strength) for weighted calculation results as the training game control parameter, with the weighting coefficients of 0.5, 0.2, 0.1, 0.1, and 0.1, respectively.

**Table 1:** Extracted motion features and physical meaning.

Number	Feature	Definition	Physical meaning
1	AMP^a^	_AMP=max(x) – max(x)_	Describes the magnitude of the movement
2	MEAN^b^	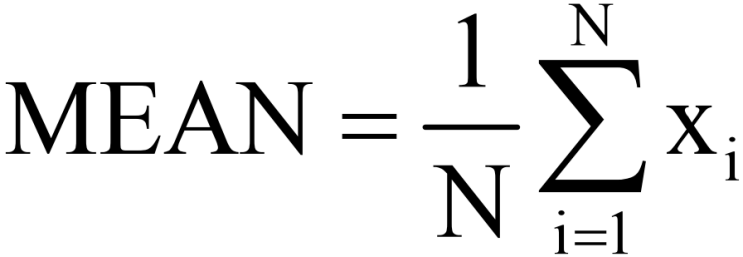	Describes the direction of the movement
3	RMS^c^	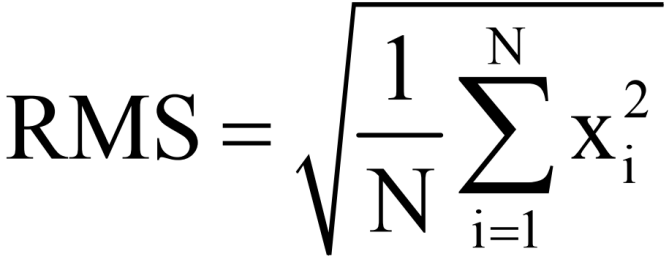	Describes motion dynamic energy
4	JERK	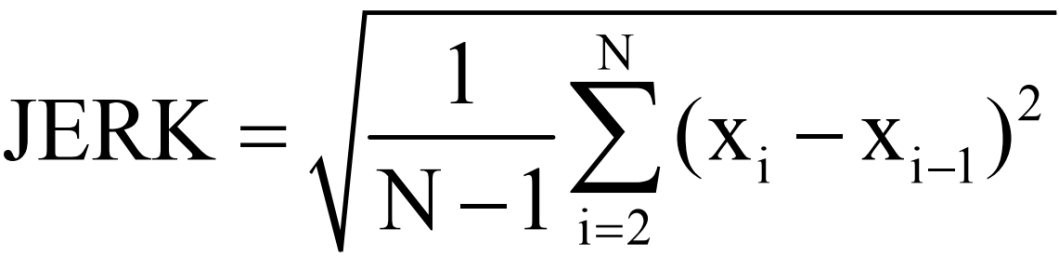	Describes motion smoothness
5	Strength	Value	Describes the magnitude of the exercise effort

^a^AMP: amplitude.

^b^MEAN: mean value.

^c^RMS: root-mean-square.

### Rehabilitation Training Software

This is a rehabilitation equipment system that integrates human-computer interaction. It uses software games to simulate daily life scenarios and guide patients in rehabilitation operation training. The computer software collects the strength, speed, distance, and other movement features of the rehabilitation training to control the game tasks, and gives feedback to the patients in a visual or auditory form, so as to guide the patients to continuously adjust their movements. Virtual games can provide clear training goals and tasks. The process of patients completing game tasks is the process of rehabilitation training. The higher the similarity between the patient’s rehabilitation training data and the standard data in terms of characteristics, the higher the patient’s score on the game task. Therefore, the training mode of human-computer interaction can greatly mobilize the enthusiasm of patients for rehabilitation training. In the absence of visual and auditory feedback, the patient is not fully aware of the abnormal movement pattern of the affected limb, often has a trunk or proximal limb compensation, and is more prone to fatigue [28].

**Figure 3:**
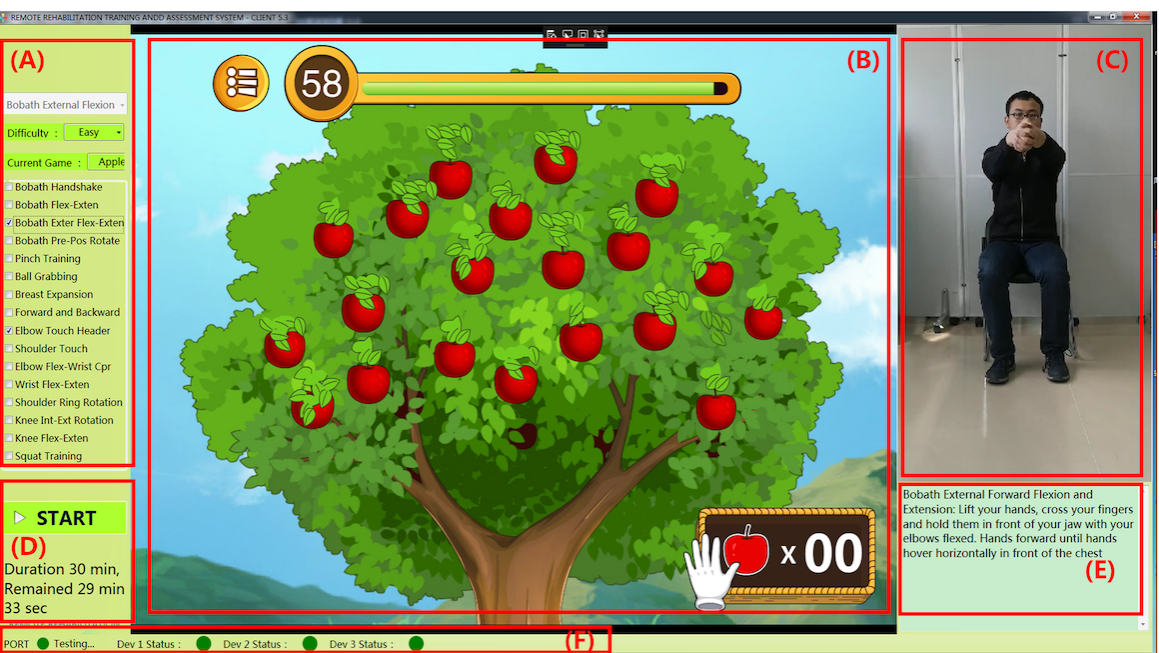
Rehabilitation training and assessment of client software: (A) rehabilitation prescription; (B) virtual game; (C) patient training; (D) exercise time; (E) action guidance; and (F) equipment operating status.

During the trial, the patient opens the Rehabilitation Training and Assessment client software (Figure 3), wears the wearable devices (2 IMU modules and rehabilitation training gloves) based on the physician’s prescription for rehabilitation training, and trains according to the standard rehabilitation video. The software can choose different games and can set different game difficulties according to the patient’s recovery status. Patients are rehabilitated by a threshold to determine whether the training action is effective or not. The thresholds are specifically 80% for difficult, 60% for moderate, and 40% for easy. By completing a valid action, the game will increase the corresponding score, and if the action is invalid, the score will remain the same. Patients will try to follow the movements of the standard training video to get a higher score during training. During the training process, the patient and the game perform human-computer interaction and receive feedback, and can simultaneously see the effect of each rehabilitation training action.

For example, in the apple picking game, each effective rehabilitation training exercise is defined as picking an apple. After the training is completed, the training score is given according to the parameters and features of the patient’s rehabilitation training, and the training data and results are automatically uploaded to the remote server so that the physician increases or decreases the length and intensity of the relevant movements according to the patient’s rehabilitation score. The new exercise prescription is automatically updated in the patient’s rehabilitation software. The clinical Fugl-Meyer score at enrollment and the game score during training were used as the basis for updating the exercise prescription. Adjustments were made once a day, and no adjustment was required for score changes of 5 points or less.

The software, games, and cloud platform included in the rehabilitation training system have been inspected by the National Medical Device Inspection Center and tested in accordance with the testing standard *GB/*t* 25000.1-2010 Software Engineering-Software Product Quality Requirements and Evaluation (SQuaRE)* [29], which meets the relevant requirements of software and network security and can be used for clinical research.

### Remote Rehabilitation Cloud Management Platform

To help physicians view and analyze the rehabilitation training situation of remote patients more timely and effectively, data are visualized through the remote rehabilitation cloud management platform, thus allowing them to manage the rehabilitation training of their patients. The remote rehabilitation management platform consists of a front-end interactive interface and a back-end data analysis system. The front-end interactive interface comprises multiple pages for users to query and edit related information. The back-end data analysis system mainly realizes the functions of analyzing multisource sensor data and generating analysis reports and pushes the evaluation results to the corresponding rehabilitation physicians.

**Figure 4:**
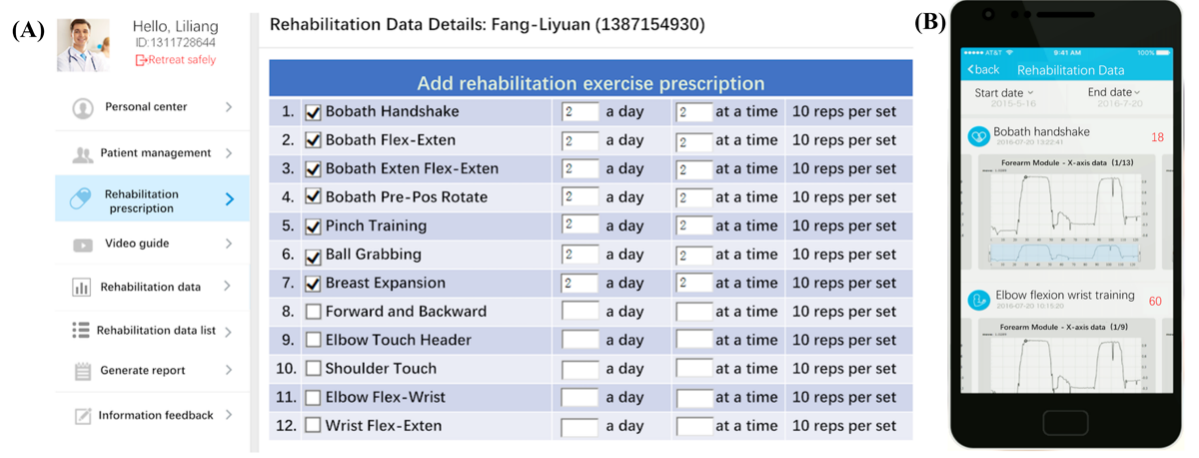
(A) Remote rehabilitation cloud management platform; (B) mobile app. Reps: repetitions.

The remote rehabilitation cloud management platform (Figure 4) includes the web terminal and the mobile app, both of which have the functions of patient information management, updating rehabilitation prescriptions, remote video guidance, viewing rehabilitation data, generating analysis reports, and constructing patient rehabilitation files to assist physicians in better managing remote rehabilitation and guiding them in rehabilitation training. Patients or their families can also log-in to the management platform to consult and communicate with rehabilitation physicians.

### Clinical Trial: Parallel Controlled 2-Center Study

#### Participants and Setting

For this pilot study, we chose patients in Tangdu Hospital and Xi’an Gaoxin Hospital with limb motor dysfunction caused by stroke 15-180 days after the onset (recovery period) and requiring rehabilitation training.

Patient inclusion criteria were as follows: (1) stroke diagnosed by computed tomography or magnetic resonance imaging within 90 days; (2) age between 30 and 75 years, male or female; (3) stable rehabilitation patients with limb motor dysfunction (with hemiplegic motor function evaluated according to the Brunnstrom upper or lower extremity grading stages II-VI) caused by stroke 15-180 days after its onset (recovery period); (4) cognition is clear and can follow the research protocol; (5) the patient can understand the study’s purpose, as well as showing sufficient compliance with the study protocol and signed the informed consent.

The following patients were excluded: (1) significant impairment of cognition and consciousness so that the Fugl-Meyer test could not be completed, (2) other significant limb lesions, such as fractures, severe arthritis, or amputation; (3) formation of limb joint contractures; (4) patients with disability, as specified by the International Classification of Functioning, Disability, and Health; (5) patients with a combination of severe primary diseases involving the cardiovascular, liver, kidney, and hematopoietic systems and mentally ill patients, as well as other circumstances that the investigator considers inappropriate to participate in this trial.

#### Experimental Design

This clinical trial is planned to be carried out in 2 clinical trial institutions at the same time and is divided into an experimental group and a control group. Patients in the experimental group received exercise training guided by the remote rehabilitation training system, routine clinical physical therapy (PT) training, and routine drug treatment. By contrast, patients in the control group received routine clinical occupational therapy (OT) training, routine clinical PT training, and routine drug treatment (see Multimedia Appendix 1 for the CONSORT [Consolidated Standards of Reporting Trials] checklist).

According to the inclusion criteria, this study selects all patients who conform to the entire trial process, that is, those who conform to the trial protocol, have good compliance, and can complete the corresponding tasks for analysis. The patients are randomly allocated to the experimental and control group in a 1:1 ratio, and the main efficacy index (Fugl-Meyer score of patients) was used as the basis for case estimation, with the sample size calculated according to the following formula [30]:


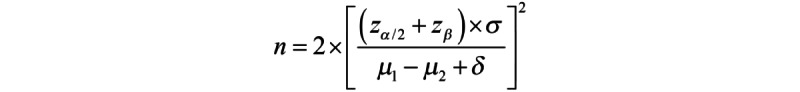


According to class II medical devices recognized by the industry, when the probability of type I error α is set to 1-sided .025, *z_α_*_/2_=1.96, and the probability of type II error β is set to .2, that is, the power (1–β=80%) is 80%, *z_β_*=0.84. According to the aforesaid formula for the number of classic cases, this study predicted that the Fugl-Meyer change value of the experimental group is μ1=11.0, the mean change of the control group is μ2=10.0, and the mean SD ()=5.5. The noninferiority margin was 40% of the mean SD, and if δ=2.2, the number of cases was calculated as n=47. Assuming a 20% dropout rate, the number of patients in each group should be at least 59. This clinical trial determined 60 cases in the experimental group and 60 in the control group, thus there were a total of 120 cases.

Considering whether the patients were exposed to PT/OT training and reducing the associated effects, this clinical trial protocol used a randomized grouping approach in which all patients who met the inclusion criteria were randomly assigned to either the test or control group according to randomization rules. The randomization method and steps were as follows: (1) Patients were randomized according to the stratified block randomization method. First, the random seed was set, then the block length was determined and stratified according to the center. SAS version 9.4 (SAS Institute) was used to generate a random grouping table of 120 patients receiving the trial (experimental group or control group). Each center was assigned consecutive random numbers that connect with each other. The patients were randomly assigned to either the experimental group or the control group according to the order in which the cases were enrolled and the randomization table.

As the training methods were different for the 2 groups, an open randomized trial was used. After randomizing patients to 1 of the 2 groups, neither the investigator nor the patients knew the grouping when baseline scoring was performed. At the beginning of the training, the randomized envelope for rehabilitation training corresponding to each randomization number was opened to know the corresponding training method. Thus, the investigator and patients only became aware of the grouping and scoring results after starting training. The statistical analysts, however, before unblinding, were not aware of the patients’ grouping.

**Figure 5:**
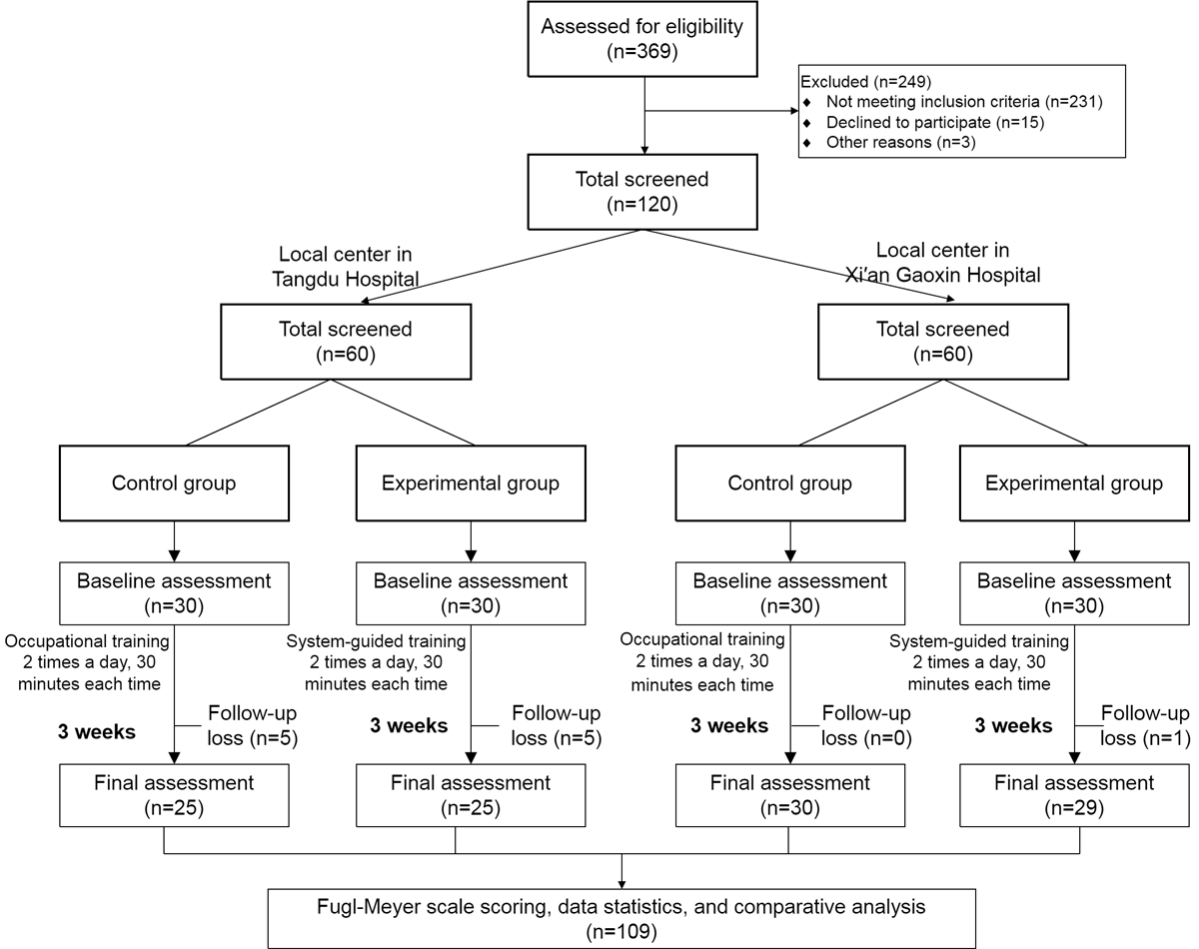
Clinical trial flowchart.

The flowchart of this clinical trial is shown in Figure 5. In the 2 rehabilitation medical centers, 369 patients were assessed for eligibility. Among these 249 were excluded, of which 231 did not meet the inclusion criteria, 15 were unwilling to participate, and 3 had other reasons. Finally, 120 patients were randomly assigned, including 60 in the experimental group and 60 in the control group. Tangdu Hospital enrolled 60 cases, including 30 in the experimental group and 30 in the control group, while Xi’an Gaoxin Hospital enrolled 60 cases, including 30 in the experimental group and 30 in the control group. However, in the actual process of the trial, according to the principle of patients’ voluntariness, 11 dropped out, and finally, a total of 109 patients completed the entire trial process.

According to related studies [28], active exercise training is more conducive to functional improvement and cortical function remodeling than passive training. According to the general rehabilitation guidelines and operating norms at home and abroad [31-33], in combination with the current commonly used clinical rehabilitation training movements and training methods, and under the advice and recommendation of many rehabilitation experts and physicians, 16 typical rehabilitation exercises were designed. The designed rehabilitation movements are used for the coordinated movement training of upper extremity, hand, and lower extremity. The training actions of the remote rehabilitation training system were as follows:

Upper extremity movements: (1) Bobath handshake training, (2) Bobath flexion and extension, (3) Bobath external anterior flexion and extension, (4) Bobath pre- and postrotation, (5) breast expansion exercise, (6) shoulder joint internal and external rotation, (7) shoulder touch training, and (8) elbow joint flexion and touch. Hand movements: (1) flexion-pressure rotation forward and backward, (2) wrist flexion and extension, (3) elbow flexion and wrist compression training, (4) finger-to-finger training, and (5) ball gripping training. Lower extremity movements: (1) squat training, (2) knee flexion and extension, and (3) knee internal and external rotation.

The experimental group adopted the training method of the remote rehabilitation training system and routine clinical PT training, whereas the control group used routine clinical rehabilitation training methods for limb motor dysfunction (ie, routine clinical OT training and routine clinical PT training).

For the inpatients in the experimental and control groups, the specific diagnosis and treatment methods were based on the condition and the test content, and the corresponding training and rehabilitation exercise methods that could be completed independently were selected in the rehabilitation hall. In addition, they performed system-guided training or routine clinical OT training 2 times a day (each session lasted 30 minutes) and conventional PT training 2 times a day (each session lasted 30 minutes). They trained no less than 10 times a week, for a total of no less than 30 times, for a total of 3 weeks.

In this study, the simplified Fugl-Meyer Motor Function Assessment scale was used for evaluation. The scale has good reliability and validity, Cronbach reliability coefficient >.80, and intraclass correlation coefficient >0.70 [34].

The scale consists of 50 items, including 33 for the upper extremities and 17 for the lower extremities, with each item rated on a scale of 0 (unable to complete the specified movement), 1 (able to partially complete), or 2 (can fully complete). The total score is 100 points. The higher the score, the better the motor function of the patient. At baseline and after 3 weeks of training, the patients were assessed by the rehabilitation physician according to the Fugl-Meyer Assessment (FMA) scale and the related results were recorded, respectively.

**Figure 6:**
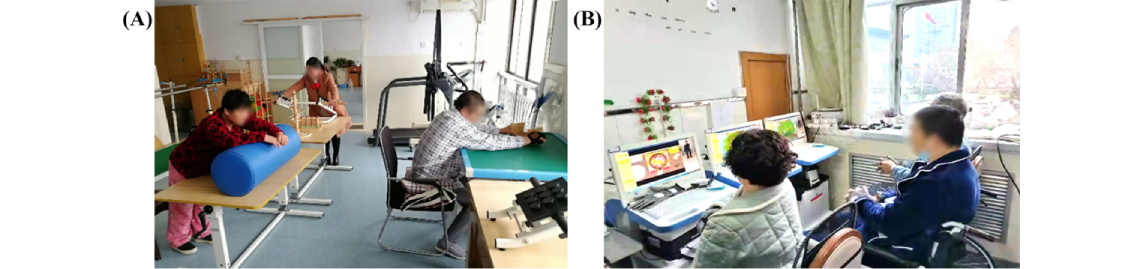
Practical application of the 2 training methods: (A) patients in the control group using conventional occupational therapy training; (B) patients in the experimental group using a remote rehabilitation training system.

To study the rehabilitation training under the real nonphysician involvement scenario, the rehabilitation physician or therapist was next to the patient during the whole rehabilitation training process, only to ensure the patient’s safety. In addition, the rehabilitation data from the experimental group were uploaded to the rehabilitation website so that the physician could view the training data and update the exercise prescription as necessary from the office. The actual training of patients with stroke is shown in Figure 6. Figure 6A shows the control group receiving conventional OT training and Figure 6B shows the experimental group wearing the wearable device and following the video and human-computer interaction game for autonomous rehabilitation training.

### Statistical Analysis

In this study, the rehabilitation status of patients with limb motor dysfunction (based on the change in the Fugl-Meyer Motor Function Rating scale score) after 3 weeks of clinical observation was used as the primary endpoint. The Fugl-Meyer score was used as the evaluation index to evaluate the clinical effectiveness of the remote rehabilitation training and evaluation system, and the safety of the system was judged by the number of adverse events and the relationship with the test system.

Descriptive statistics were used in this study to characterize demographic parameters and other baseline characteristic values. In this pilot study, a total of 109 patients ultimately completed the full trial, and statistical analyses and discussions of the data were conducted for these patients.

For descriptive statistics, demographic data, and other baseline characteristic values, parametric analysis was performed using targeted statistical methods, and the *P* value of inferential statistics was listed as the descriptive result. For the change in Fugl-Meyer score from baseline to 3 weeks of treatment, the difference between the 2 groups and its bilateral 95% CI were calculated.

SAS version 9.4 was used for analysis in this study. All statistical tests were 2-sided, and a *P* value ≤.05 was considered statistically significant.

### Ethics Approval

This study was approved by the Ethics Committees of Tangdu Hospital (approval number 201912-08) and Xi’an Gaoxin Hospital (2020 ethics review number 001). All patients participating in this study have signed the informed consent form.

## Results

### Baseline Data Analysis

The statistical results of demographic parameters and other baseline characteristic values are presented in Table 2. Different parameters of the experimental and control groups were statistically analyzed by different statistical methods. There was no significant difference in age (*P*=.81), BMI (*P*=.39), systolic blood pressure (*P*=.25), and diastolic blood pressure (*P*=.41) between the 2 groups by (1-sided) *t* test (*P*>.05).

**Table 2:** Analysis of demographic parameters of patients with stroke (n=60).

Characteristics	Control group				Experimental group				
	Values^a^	95% CI (lower-upper)	Range (median)	Values^a^		95% CI (lower-upper)	Range (median)	*P* value	
Age (year)	55.82 (9.68)	53.32-58.32	34.00-73.00 (55.00)	56.25 (9.83)		53.71-58.79	33.00-73.00 (56.00)	.81	
Gender (male)	43 (71.67)			43 (71.67)				>.99	
Course of disease (days)	61.20 (46.67)	49.15-73.25	15.00-169.00 (41.00)	46.22 (36.63)		36.75-55.68	15.00-165.00 (33.00)	.15	
Systolic blood pressure (mmHg)	127.47 (11.56)	124.48-130.45	96.00-151.00 (127.00)	130.13 (13.73)		126.59-133.68	92.00-166.00 (130.00)	.25	
Diastolic blood pressure (mmHg)	81.05 (9.00)	78.73-83.37	60.00-105.00 (80.00)	82.45 (9.51)		79.99-84.91	57.00-107.00 (80.50)	.41	
BMI (kg/m^2^)	24.68 (4.08)	23.59-25.76	18.34-44.92 (23.70)	24.66 (2.59)		23.99-25.33	18.34-44.92 (24.57)	.39	
Stroke type (cerebral infarction)	37 (61.67)	—^b^	—	32 (53.33)		—	—	.36	
Hypertension	44 (73.33)	—	—	43 (71.67)		—	—	.84	
Hyperlipidemia	7 (11.67)	—	—	4 (6.67)		—	—	.53	
Arteriosclerotic coronary disease/myocardial infarction	10 (16.67)	—	—	12 (20.00)		—	—	.64	

^a^Data are mean (SD) or n (%).

^b^Not applicable.

Although the mean course of stroke in the 2 groups was 61.20 and 46.22, respectively, in the Wilcoxon test for these 2 nonnormally distributed data, *P*=.15 (*P*>.05), indicating that there was no statistically significant difference between the 2 groups. The reason for the difference in the means of the 2 groups was that there were 2 cases in the control group with stroke duration days close to 180 days, which increased the mean, but did not affect the overall experimental results.

The chi-square test showed that there was no significant difference between the 2 groups in gender (*P*>.99), stroke type (*P*=.36), hypertension (*P*=.84), and arteriosclerotic coronary disease/myocardial infarction (*P*=.64). Fisher test showed that there was no significant difference in hyperlipidemia between the 2 groups (*P*=.53). These statistical results showed that in terms of various parameters, there was no statistical difference between the control group and the experimental group.

### Results of the Clinical Trial

At baseline and 21 days, patients in the experimental group and patients in the control group were evaluated for motor function according to the FMA scale by experienced clinical rehabilitation physicians. The results of the experimental and control groups were statistically analyzed using the *t* test, and the relevant results are presented in Table 3. A total of 55 patients (92%) in the control group completed all trials, whereas a total of 54 patients (90%) in the experimental group completed all trials. Physician Fugl-Meyer mean total score changes in the control group were 11.98 (SD 8.46; 95% CI 9.69-14.27), whereas those in the experimental group were 17.56 (SD 11.65; 95% CI 14.37-20.74; *P*=.005). Physician Fugl-Meyer mean upper extremity score changes in the control group were 7.45 (SD 7.24; 95% CI 5.50-9.41), whereas those in the experimental group were 11.28 (SD 8.59; 95% CI 8.93-13.62; *P*=.01). Physician Fugl-Meyer mean lower extremity score changes in the control group were 4.53 (SD 4.42; 95% CI 3.33-5.72), whereas those in the experimental group were 6.28 (SD 5.28; 95% CI 4.84-7.72; *P*=.06).

**Table 3:** Statistical analysis of physician scores according to the Fugl-Meyer scale.

Analysis			Control group							Experimental group							*P* value
			Mean (SD)		95% CI (lower-upper)		Range (median)		Mean (SD)			95% CI (lower-upper)		Range (median)			
**Total score**																	
	0 days	41.11 (27.49)		33.68-48.54		8.00 to 96.00 (35.00)		43.28 (25.09)			33.68-48.54		13.00 to 97.00 (35.50)		.67		
	21 days	53.09 (28.40)		45.41-60.77		8.00 to 99.00 (48.00)		60.83 (23.80)			54.34-67.33		20.00 to 98.00 (60.00)		.13		
	21 to 0 days	11.98 (8.46)		9.69-14.27		0.00 to 39.00 (10.00)		17.56 (11.65)			14.37-20.74		1.00 to 49.00 (14.00)		.005		
**Upper extremity**																	
	0 days	23.67 (19.93)		18.28-29.06		4.00 to 65.00 (16.00)		25.48 (18.69)			20.38-30.58		4.00 to 64.00 (19.50)		.63		
	21 days	31.13 (21.67)		25.27-36.99		4.00 to 66.00 (25.00)		36.76 (18.65)			31.67-41.85		7.00 to 66.00 (36.50)		.15		
	21 to 0 days	7.45 (7.24)		5.50-9.41		0.00 to 36.00 (5.00)		11.28 (8.59)			8.93-13.62		0.00 to 32.00 (9.50)		.01		
**Lower extremity**																	
	0 days	17.44 (9.36)		14.91-19.97		4.00 to 34.00 (17.00)		17.80 (8.03)			15.61-19.99		5.00 to 33.00 (16.50)		.83		
	21 days	21.96 (8.36)		19.70-24.22		4.00 to 34.00 (23.00)		24.07 (7.04)			22.15-26.00		10.00 to 34.00 (26.00)		.16		
	21 to 0 days	4.53 (4.42)		3.33-5.72		–7.00 to 16.00 (4.00)		6.28 (5.28)			4.84-7.72		–3.00 to 22.00 (5.00)		.06		

**Figure 7:**
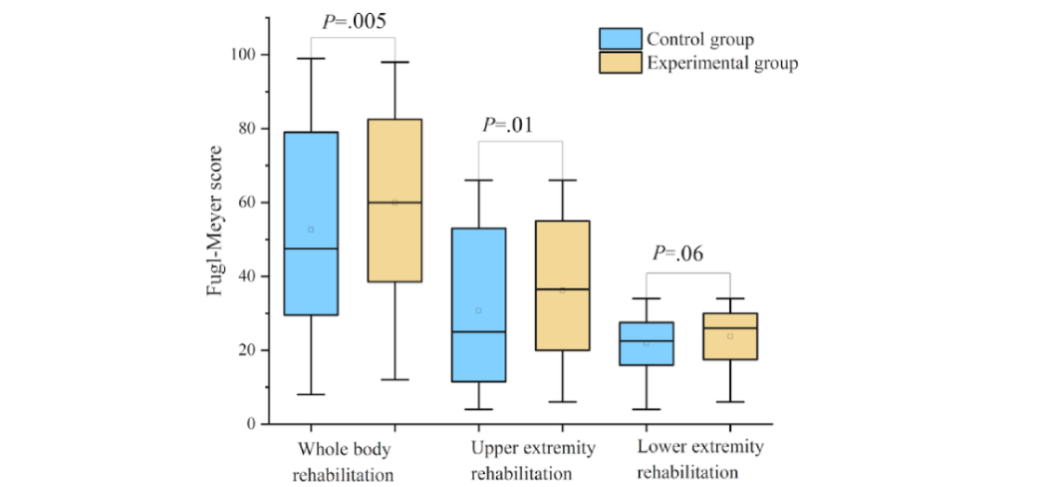
Fugl-Meyer Scale score results of the control group and the experimental group.

Figure 7 shows the change distribution of Fugl-Meyer scores in the control group and the experimental group after 21 days of rehabilitation training, including the total score, upper limb score, and lower extremity score. The test results showed that the experimental group was better than the control group in the improvement of the total score, upper limb score, and lower extremity score. In the general evaluation and upper limb rehabilitation training, there were significant differences in the changes between the 2 groups (*P*=.005 and .01, respectively), and there was no significant difference in the changes in the lower extremity score between the 2 groups (*P*=.06). The reason may be that, on the one hand, there are only 3 lower extremity rehabilitation exercises, and on the other hand, because the patients also undergo exercise rehabilitation training for the lower extremities in daily walking and other activities, there is no significant difference in the lower extremity rehabilitation effects between the 2 groups (*P*=.06).

**Figure 8:**
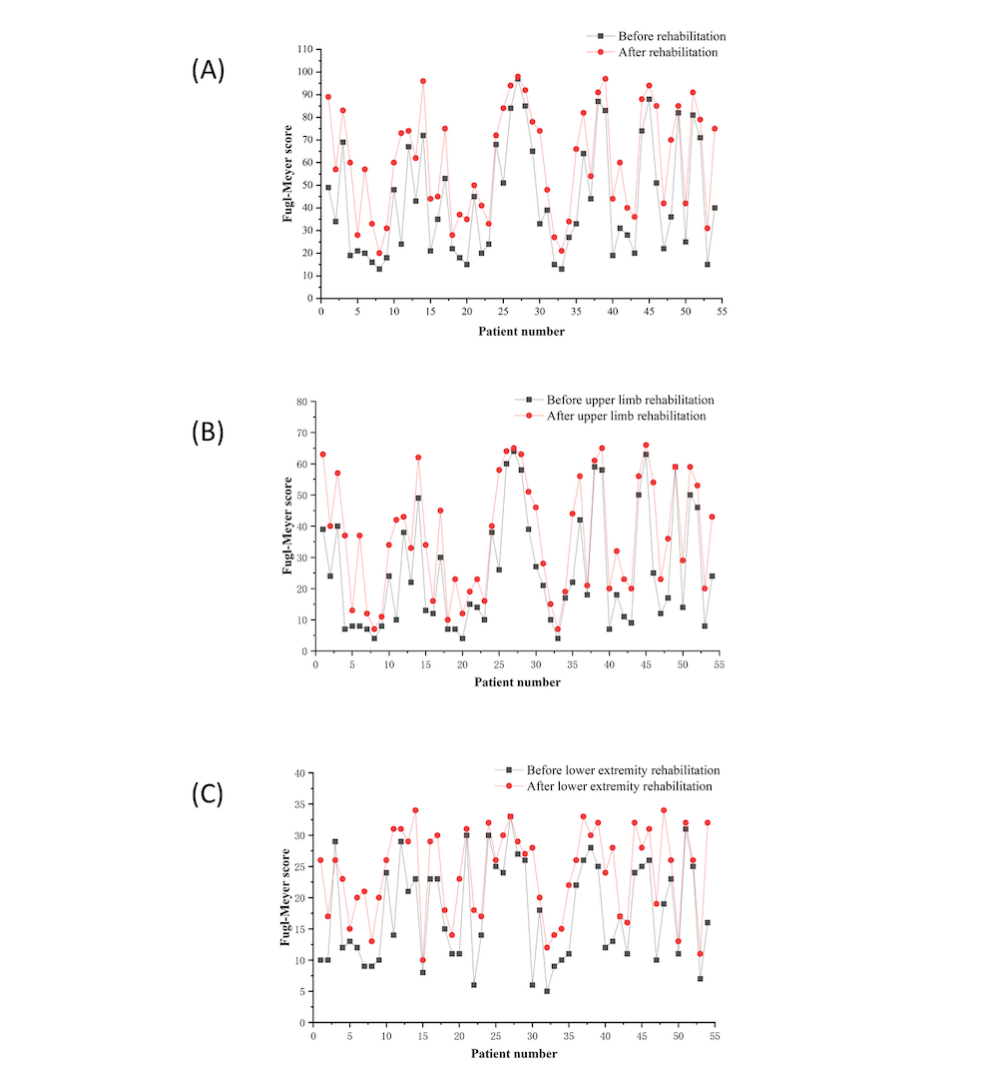
(A) Comparison of total scores of the Fugl-Meyer Scale before and after rehabilitation of patients in the experimental group; (B) comparison of Fugl-Meyer Scale upper limb scores of patients in the experimental group before and after rehabilitation; (C) comparison of Fugl-Meyer Scale lower extremity scores before and after rehabilitation in the experimental group.

To analyze the effect of a single patient’s use of a remote rehabilitation training system on motor function recovery, this study compared the results of the total score, upper limb score, and lower limb score of all patients in the experimental group before and after rehabilitation, as shown in Figure 8. Combined with data in Table 3, the average score of patients before rehabilitation was 43.28, and the average score after rehabilitation training was 60.83, with an average increase of 17.56. The average score of upper limbs before rehabilitation was 25.48, and the average score of upper limbs after rehabilitation training was 36.76, with an average increase of 11.28. The average score of the front lower extremity was 17.80, and the average score of the lower extremity after rehabilitation training was 24.07, an average increase of 6.28. These results show that for all patients using the remote rehabilitation training system, after 21 days of rehabilitation training, the FMA total score, the upper limb score, and the lower extremity score have improved significantly, that is, the patient’s exercise ability has been effectively recovered.

**Figure 9:**
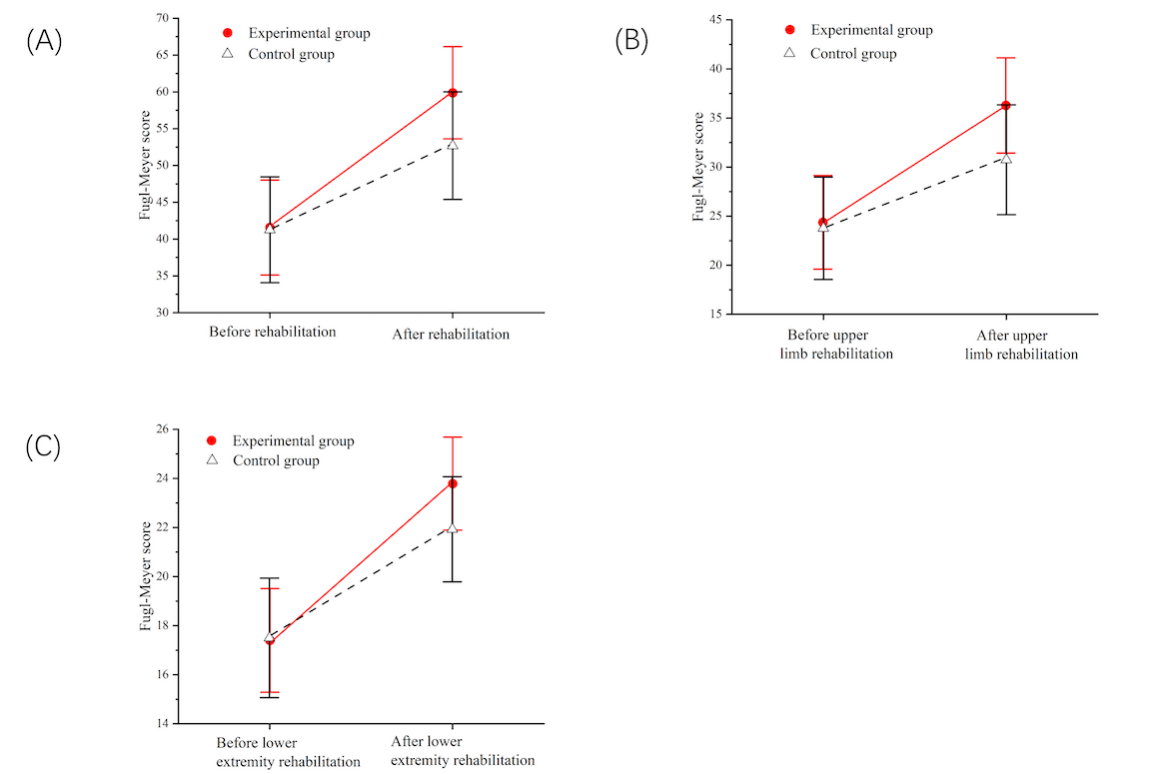
(A) Changes in the total score of different rehabilitation methods in the control group and the experimental group; (B) changes in upper limb scores in different rehabilitation methods in the control group and experimental group; (C) changes in lower extremity scores in different rehabilitation methods in the control group and experimental group.

To compare the effects of using the remote rehabilitation training system and receiving conventional OT training on the recovery of patients’ exercise ability, this study compared the control group and the experimental group before and after rehabilitation, as shown in Figure 9. Compared with receiving conventional OT training, the patient’s exercise ability improved significantly through the remote rehabilitation training system, and the difference was significant (*P*=.005; Table 3). Among them, the Fugl-Meyer score change value of the upper limb was greater than that of the lower extremity, and all patients using the remote rehabilitation training system had a better rehabilitation effect on the upper limb remote rehabilitation.

Finally, adverse events in this trial were analyzed. Adverse events are unfavorable medical events that occur during a clinical trial, whether related to a device or not. During the entire clinical trial, 28 adverse events were reported in the control group, with an incidence rate of 46.67%, and 22 adverse events in the experimental group, with an incidence rate of 36.67%; however, there was no significant difference between the 2 groups (*P*=.27). The adverse events that occurred were judged by the investigator to be irrelevant to this test system.

## Discussion

### Principal Findings

In this study, aiming at the rehabilitation training of patients with limb movement dysfunction such as stroke, a number of wireless wearable devices were developed based on IMU inertial device and bending sensor. Using the Zigbee wireless networking technology, the movement data from patients’ rehabilitation training can be collected at the same time. Through data fusion and signal processing, real-time rehabilitation training exercise monitoring and exercise ability analysis are realized. Using rehabilitation training games based on daily life scenes, human-computer interaction rehabilitation training is realized. Further, the patient’s rehabilitation training data and results are recorded and uploaded to the remote server platform, so that the remote-end rehabilitation physician can view, analyze, and guide the patient to undergo effective rehabilitation training in a timely manner and improve the patient’s enthusiasm and compliance for rehabilitation training.

In addition, the game scenes correspond to the rehabilitation training actions, and the actions and games are matched according to the parts of the patient’s body that need rehabilitation. At the same time, the patient can modify the default game and the system will automatically save the record of the game selected by the patient and use it for subsequent rehabilitation training. With the improvement of the patient’s exercise ability, the difficulty of training will increase, and the rehabilitation exercise prescription will become more diversified. Presenting continually challenging new tasks helps patients stay motivated and interested in rehabilitation therapy. The virtual training scene based on daily life can reduce the danger caused by the wrong operation of patients with stroke in the real environment.

Based on the clinical trial of 109/120 (90.8%) patients with stroke, those in the experimental and control groups were scored according to the FMA scale at baseline and 21 days, respectively, and the scores of the 2 groups were compared and statistically analyzed. The results showed that the experimental group outperformed the control group in terms of changes relative to baseline in Fugl-Meyer total scores, upper extremity scores, and lower extremity scores, and that the patients’ upper and lower extremity motor abilities were better restored and improved, with significant improvement in upper extremity and total scores and some improvement in lower extremity scores. Other studies also found that lower extremity training improved motor function [35,36]. This clinical trial shows that the remote rehabilitation training system is used for the rehabilitation training of limb motor function of patients with stroke, and that the effect is better than that of routine clinical OT training.

In addition, in terms of safety, no adverse events related to this system occurred during the entire trial. Therefore, the designed remote rehabilitation training system based on wearable devices and human-computer interaction is used for rehabilitation training of patients with stroke and other limb motor dysfunction, which has good efficacy and good safety.

### Comparison With Prior Work

According to the literature [37], long-term and specific rehabilitation training can maximize the recovery of patients’ health and confidence. However, patients are less willing to participate in rehabilitation programs for daily repetitive and passive training [38,39]. By contrast, active training of patients is more effective than passive training and can enhance patient outcomes [40,41].

Chae et al [42] proposed a smartwatch and machine learning–based remote rehabilitation system for home training of patients’ upper limbs. However, the number of patients was small, and the system is only for upper limb training; besides, the actual accuracy of home motion detection was not evaluated. Held et al [43] proposed a method of gait rehabilitation for patients with stroke, combining mobile augmented reality technology and sensor technology to adjust and train patients to walk. However, the device requires set up and calibration, making it more difficult for patients to use. The use of robotic technology for the rehabilitation of patients with stroke has been greatly developed. Ren et al [13] developed a wearable ankle joint rehabilitation robot to perform active and passive training on patients, but only for patients with acute stroke requiring ankle rehabilitation. Zhang et al [44] designed a desktop rehabilitation robot to train and evaluate the motor function of the upper limbs of patients.

Most experimental systems are complicated to use, expensive, inconvenient for patients to perform home training, and have few training movements, and therefore, they cannot undergo comprehensive training for the whole body. In addition, most of the aforesaid studies were performed under the supervision of physicians on-site, and cannot be applied in remote environments such as in home.

In similar clinical trials of the efficacy of home remote rehabilitation, Cramer et al [45] conducted a comparison trial with clinical rehabilitation modalities for patients with stroke having upper extremity motor deficits and showed that activity-based training significantly improved arm motor function, but the trial was only for upper extremity and lacked further analysis for patients requiring lower extremity rehabilitation. In a trial comparing lower extremity rehabilitation, Kang et al [46] compared patients’ activities of daily living abilities through treadmill training, and reported that the Nordic treadmill training was an effective aid. However, the trial was performed for patients with mild issues under the supervision of the therapist, and there was no random allocation method, so caution should be exercised when interpreting the findings. In terms of human-computer interaction, Lee’s study [35] found that mobile phone–based virtual reality applied to patients’ stationary bicycle training improved lower extremity motor function recovery, but the movement of both legs was easily dominated by the healthy side of the body and lacked targeted training for the affected side of the body.

Therefore, the wearable remote rehabilitation training system for patients with stroke designed in this study can effectively overcome the aforesaid technology problems. Besides, the system was further designed and optimized based on the previous versions. Consequently, patients can receive effective training and guidance at home or in the community. In addition, the effectiveness and safety of the designed stroke active rehabilitation training system were verified by analyzing the results of the finalized clinical trial of 109 patients with stroke.

### Limitations and Prospects

During the clinical experiment, almost all patients in the experimental group and rehabilitation physicians expressed strong interest in the designed rehabilitation training system owing to wearable devices and human-computer interaction training games.

However, according to the recommendations of rehabilitation physicians and patients, the system still has some limitations and needs further improvement for its better application in remote and home environments. In future work, the following improvements will be made.

First, according to the patients’ suggestion, the size/resolution of the standard training video on the software interface needs to be increased, with the action details and precautions also displayed, to facilitate the patient to standardize the rehabilitation training according to the standard video. Second, the software needs to have built-in instructions and videos on how to wear the wearable device so that patients who are unfamiliar with the system can adapt more quickly and actively participate in rehabilitation training. Third, we need to add more rehabilitation training actions and more human-computer interaction sports games in daily life scenarios to meet the needs for more refined and diversified rehabilitation training.

In terms of the experimental design, the following limitations apply:

1. The pilot was set up in a hospital rehabilitation hall rather than in a remote and decentralized home setting to more fully assess the effectiveness of patient rehabilitation training and the overall management of the rehabilitation process.

2. Considering that hospital patients usually recover in the hospital for about 3 weeks, the trial was shorter than other studies [47,48]. After the trial, only 3 months of telephone follow-up was conducted for patients, and no abnormalities related to the trial were found. The clinical follow-up results are not taken into account in this study, which is one of the limitations of the design scheme of this study.

3. This trial only studied the patients’ performance in the FMA scale. The follow-up research will include the Activities of Daily Living scale, the Wolf Motor Function Test, the patients’ psychological status and satisfaction level, the impact of stroke publicity and education, among others, to further explore the rehabilitation effect of the remote rehabilitation training system.

### Conclusions

This study found that the use of the remote intelligent rehabilitation training system designed based on wearable devices and human-computer interaction training tasks has a significant effect on the rehabilitation of motor function of patients with stroke, which can replace routine clinical OT training and improve the motivation, compliance, and rehabilitation effect of the training. In the future, improvements to the system will be made based on physician and patient recommendations, and through the medical device registration certification, it will be used without the participation of physicians or therapists, such as in rehabilitation training halls, and in remote environments, such as communities and homes.

## Appendix

Multimedia Appendix 1 CONSORT-eHEALTH checklist (V 1.6.1).

## Abbreviations

AMP: amplitude
FMA: Fugl-Meyer Assessment
IMU: inertial measurement unit
MEAN: mean value
OT: occupational therapy
PT: physical therapy
RMS: root-mean-square
SQuaRE: Software Engineering-Software Product Quality Requirements and Evaluation
